# Deuterated Drugs and Biomarkers in the COVID-19 Pandemic

**DOI:** 10.1021/acsomega.2c04160

**Published:** 2022-11-13

**Authors:** Ross D. Jansen-van Vuuren, Luka Jedlovčnik, Janez Košmrlj, Thomas E. Massey, Volker Derdau

**Affiliations:** † Faculty of Chemistry and Chemical Technology, University of Ljubljana, Večna pot 113, Ljubljana 1000, Slovenia; ‡ Department of Chemistry, Queen’s University, 90 Bader Lane, Kingston, Ontario K7L 3N6, Canada; § Department of Biomedical and Molecular Sciences, School of Medicine, Queen’s University, Botterell Hall, 18 Stuart Street, Kingston, Ontario K7L 3N6, Canada; ∥ Research & Development, Integrated Drug Discovery, Isotope Chemistry, Sanofi-Aventis Deutschland GmbH, Industriepark Höchst G876, Frankfurt/Main 65926, Germany

## Abstract

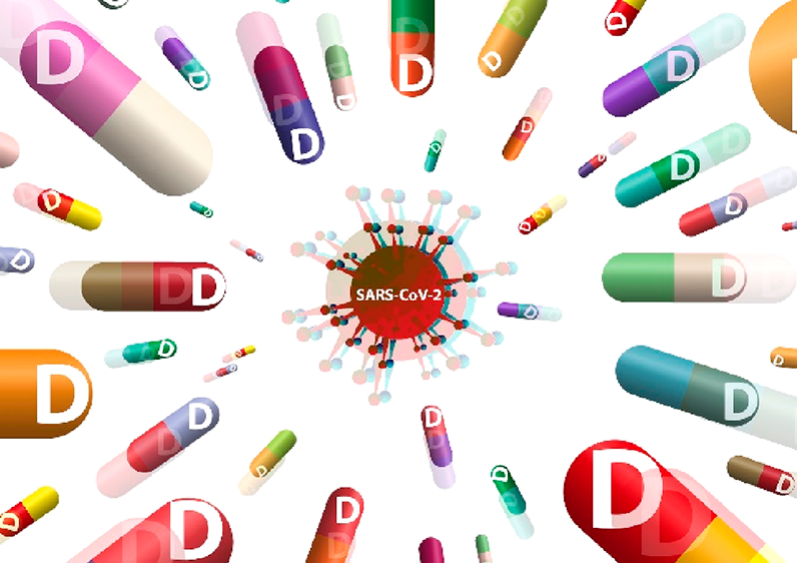

Coronavirus disease 2019 (COVID-19) is a highly contagious disease caused by the severe acute respiratory syndrome coronavirus 2 (SARS-CoV-2). Initially identified in Wuhan (China) in December 2019, COVID-19 rapidly spread globally, resulting in the COVID-19 pandemic. Carriers of the SARS-CoV-2 can experience symptoms ranging from mild to severe (or no symptoms whatsoever). Although vaccination provides extra immunity toward SARS-CoV-2, there has been an urgent need to develop treatments for COVID-19 to alleviate symptoms for carriers of the disease. In seeking a potential treatment, deuterated compounds have played a critical role either as therapeutic agents or as internal MS standards for studying the pharmacological properties of new drugs by quantifying the parent compounds and metabolites. We have identified >70 examples of deuterium-labeled compounds associated with treatment of COVID-19. Of these, we found 9 repurposed drugs and >20 novel drugs studied for potential therapeutic roles along with a total of 38 compounds (drugs, biomarkers, and lipids) explored as internal mass spectrometry standards. This review details the synthetic pathways and modes of action of these compounds (if known), and a brief analysis of each study.

## Introduction

### Drug Development and Discovery for COVID-19

COVID-19 is primarily characterized as an acute respiratory illness caused by a droplet-borne coronavirus, SARS-CoV-2, also an RNA virus. By August 2022, the COVID-19 pandemic had resulted in almost 600 million infections, ∼6.4 million deaths, major disruption to global trade and travel, and closure of local businesses. While most patients experience a mild to moderate respiratory infection and fever, and can recover without the need for special treatment, for some, COVID-19 is more severe, leading to major respiratory shutdown and multiple organ failure, requiring intensive care. Immunocompromised people are at greater risk of experiencing severe COVID-19 symptoms or death, while their immune response to vaccination is not as strong as in nonimmunocompromised people.

While vaccination has offered the most effective way to avoid experiencing a serious case of COVID-19, treatment is still needed to reduce the symptoms and hasten the healing process. Two major approaches that have been taken to discover drugs that could be used to treat COVID-19 include the repurposing of known drugs^1^ and the development of novel drugs.^2^ Both types of drugs are proposed to act either by disrupting a certain component of the life cycle of the coronavirus^3^ or as anti-inflammatories,^4^ altering the body’s response to the virus. Currently, several treatments are FDA approved,^5^ while much research (including clinical trials) is underway to demonstrate the efficacy of treatments against COVID-19.^6^ In seeking a potential treatment for COVID-19, deuterated drugs (compounds in which some hydrogens have been exchanged for deuterium) have been explored either as therapeutic agents or as internal MS standards for studying the pharmacological properties of new drugs by quantifying the parent compound and possible metabolites by liquid chromatography/mass spectrometry (LC/MS) assays. To date, no work has been carried out to review these deuterated compounds. Thus, this paper offers a comprehensive review of all deuterated compounds explored as internal standards, potential treatments, or biomarkers during the development of treatment for COVID-19 along with their synthetic pathways and modes of action (where this is known).

Since the FDA approval of the first deuterated drug in 2017,^7,8^ there has been a major surge of interest in the development of new deuteration methodologies and the preparation of novel deuterium-labeled compounds.^9^ The incorporation of deuterium has been found to overcome drug limitations related to toxicity, bioavailability, and pharmacokinetics, mostly by altering the metabolic profile of the drug of concern.^10,11^ Also, the formation of deuterated compounds as internal standards for analytical purposes is advantageous due to the availability and economical value of deuterium over other isotopes such as ^15^N or ^18^O.

By collating studies involving deuterated compounds related to COVID-19, this review seeks to be an easy reference tool for practitioners of isotope exchange chemistry as well as for medicinal chemists, especially those involved in the development of either COVID-19 treatments or methods to study the efficacy of such therapies. By illuminating the approaches taken in the synthesis and applications of deuterated drugs and biomarkers, we hope to inspire new ideas for deuterium-labeled compounds beyond what is in the literature.

### Deuterated Compounds

Deuterium (D) is an isotope of hydrogen (H), having the same proton number but double the mass due to a neutron in the nucleus. Thus, exchanging H for D results in a slight increase in the activation energy (EA) needed for bond cleavage (1.4 kcal/mol) as well as a lower reaction rate compared with C–H (C–D has a lower zero-point energy), an effect known as the primary deuterium kinetic isotope effect (DKIE) and expressed as *k*_H_/*k*_D_, the ratio of the reaction rate constants of C–H versus C–D bond cleavage.^12^ Although the EA value seems negligible, especially considering the nearly constant body temperature of 37–39 °C, it has been shown to alter the metabolic profiles of compounds whose metabolic pathways are dependent on C–H bond cleavage.^11^ Examples include drugs metabolized by cytochrome P450s or aldehyde oxidases, deuteration of which can result in pharmaceutical compounds with improved pharmacokinetics and reduced toxicity^13^ but equal potency to the parent drug.^11,14^ However, some deuterated drugs provide no improvement in terms of the metabolic process, while others are prone to unexpected metabolic switching resulting in the deuterated analogue having no pharmacokinetic advantages over the parent compound.^15^

Since deuterated drugs are identical spatially and have the same charge distribution as their nondeuterated analogues, in most cases, both share similar physiochemical properties, e.g., lipophilicity,^16^ and therefore interact comparably with cellular components such as enzymes, ion channels, receptors, and transporters.^7,17^ However, a few studies have shown that deuterium labeling can have a complex effect on intermolecular interactions^18^ and binding to enzymes,^19^ while other studies, particularly focused on histamine receptors, have questioned the fact that labeling retains the same interactions with the target compared to the protium-containing compound.^20^

In this paper, we will review deuterated drugs that have been explored as possible therapeutics for COVID-19 as well as those which have been used as internal MS standards for probing the properties of new or repurposed drugs being explored as treatment options for COVID-19.

## Deuterated Drugs as Therapeutics for the Treatment of COVID-19

Some drugs that have demonstrated efficacy against SARS-CoV-2 require relatively high dosages and/or result in patients experiencing adverse effects. This is of particular concern since patients suffering from COVID-19 already have compromised immune systems. For some COVID-19 antiviral and anti-inflammatory drugs, toxicity is attributed to the parent compound, while in some cases, reactive metabolites are implicated. Furthermore, COVID-19 causes inflammation of the liver, resulting in suppression of the hepatic cytochrome P450 enzymes, causing reduced drug clearance, higher plasma drug concentration, and severe toxicity in some cases, triggering further deterioration in the condition of COVID-19 patients.^21^ The potential for toxicity of COVID-19 antivirals is amplified by both the presence of a complex disease and the fact that multiple drugs are often used concurrently.^22^ For example, cardiac dysfunction attributable to COVID-19 can be exacerbated by some COVID-19 drugs.^23^ Furthermore, drug–drug interactions at the level of drug disposition, including metabolism and transport, could increase plasma concentrations of certain drugs and their metabolites, further increasing cardiac risk.

**Figure 1:**
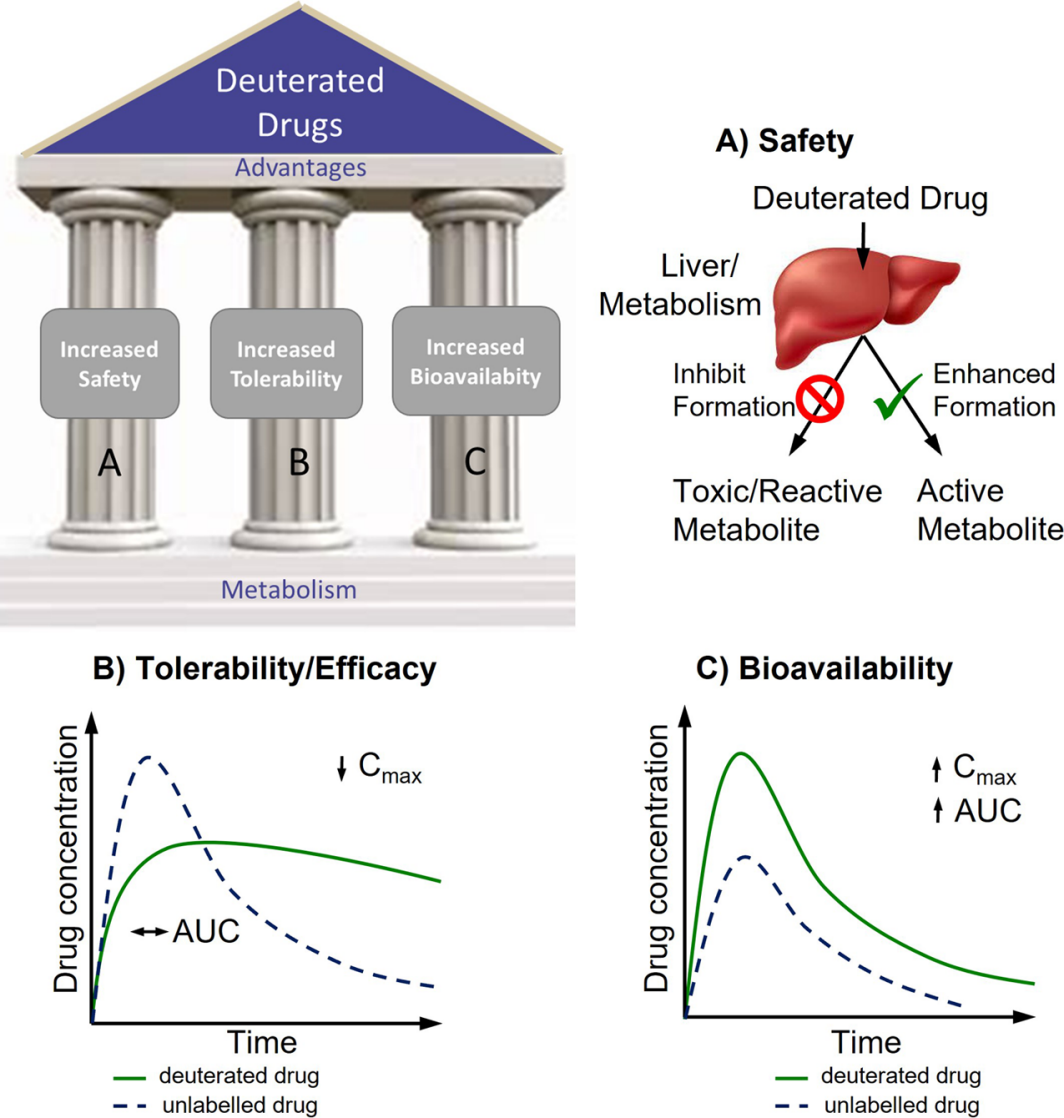
Three main advantages potentially provided by deuterated drugs: increased (A) safety, (B) tolerability, and (C) bioavailability. These are achieved, respectively, by (A) “metabolic shunting”, resulting in reduced exposure to undesirable (toxic or reactive) metabolites, (B) reduced systemic clearance, resulting in increased half-life, and (C) first-pass metabolism, resulting in higher bioavailability of the nonmetabolized drug. AUC is area under the curve and represents drug exposure over time; *C*_max_ is the maximum or peak concentration of a drug. Adapted with permission from ref (24). Copyright 2014 The Pharmaceutical Society of Japan.

**Figure 2:**
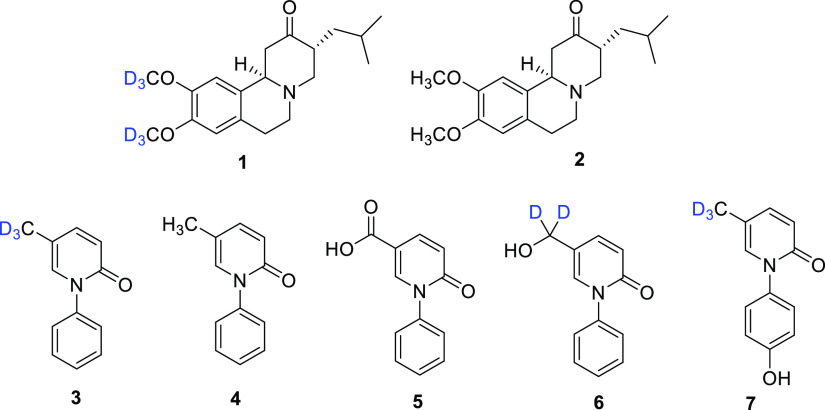
Chemical structures of deutetrabenazine (**1**), tetrabenazine (**2**), deupirfenidone (**3**), and pirfenidone (**4**). Also shown are compound **5**, the primary metabolite of both **3** and **4**, and compounds **6** and **7**, the secondary metabolites of **3**.

Deuteration presents an opportunity to improve three major characteristics of drugs: (i) the safety of the drug via “metabolic shunting” (the generation of less toxic metabolites), (ii) higher drug tolerability, which means that the drug can be administered in lower dosages and that it remains at a more constant blood plasma concentration (rather than having pronounced peaks and troughs), and (iii) increased drug bioavailability (Figure 1).^24^ This has invigorated research into the deuteration of known drugs which have been removed from clinical studies or circulation due to safety concerns, resulting in the recent approval of the first deuterated drug for commercial use by the U.S. Food and Drug Administration (FDA). This drug is deutetrabenazine (**1**), the deuterated version of tetrabenazine (**2**) (Figure 2), used for the treatment of Huntington’s disease, an involuntary movement disorder.^25^

In the case of **1**, exchanging the six methoxy protiums with deuteriums slightly alters the metabolism of the drug, increasing its safety and tolerability by conferring upon it an extended half-life and a more stable plasma concentration.^7^ As no head-to-head trial with tetrabenazine has ever been performed, it is still not clear if there is a significant safety advantage. However, the recommended daily dosage of tetrabenazine is approximately double the recommended starting dosage of deutetrabenazine.^26^

Deuterated versions of various medicinal compounds are now being explored as treatments; as of 2019, there were more than 20 deuterated drugs in clinical trials, with 6 having reached Phase III, while 200–300 patents had been filed for deuterated medicinal compounds.^11^ Several deuterated drugs were explored as potential therapeutics for COVID-19. These compounds are isotopologues (i.e., molecules that only differ from the parent molecule in their isotopic composition) of either repurposed drugs or completely new drugs. These compounds are reviewed in the following section.

### Repurposed Drugs

#### Deupirfenidone (LYT-100)

Deupirfenidone or LYT-100 (**3**, CAS No. 1093951-85-9) is the deuterated form of pirfenidone (**4**) (Figure 2), originally used for the treatment of idiopathic pulmonary fibrosis, a severe lung disease.^27^ LYT-100, developed by PureTech Health (based in Boston) and currently in Phase 2 clinical trials (ClinicalTrials.gov Identifier NCT04652518), was already under consideration to target lymphedema (lung inflammation) and lung fibrosis prior to the pandemic and was therefore ideally suited to treat patients suffering from long COVID-19.^27,28^ Metabolism of pirfenidone is carried out by cytochrome P450 (CYP1A2) and involves oxidation of the methyl group at C-5 of the pyridin-2(1*H*)-one ring of **4**, leading to the formation of the primary metabolite, 5-carboxy-pirfenidone (**5**), which is inactive compared with **3** or **4**. Thus, replacing hydrogen with deuterium on the methyl group ought to inhibit the metabolism of the drug and enable less frequent dosing, and this was shown to be the case through a 2021 Phase 1 clinical trial (ClinicalTrials.gov Identifier NCT04243837).^29^ When **3** undergoes metabolism, the secondary metabolites 5-hydroxymethylpirfenidone-*d*_2_ (**6**) and 4′-hydroxypirfenidone-*d*_3_ (**7**) are formed in low concentrations.^28^

**Scheme 1:**
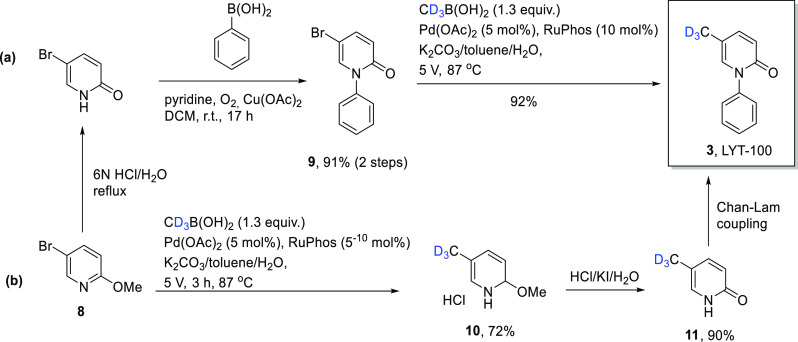
Suzuki-Coupling Approaches to the Synthesis of Deupirfenidone (**3**): (a) Methylation-Last Route and (b) Methylation-First Route

Deupirfenidone (pirfenidone-*d*_3_) (**3**) can be prepared via many routes, mostly involving the use of alkyllithiums at cryogenic temperatures.^30,31^ More recently, however, three routes have been reported which employ milder conditions and have higher yields. First, **3** can be prepared via visible light (390 nm) driven, TBADT/thiol-catalyzed deuterium labeling in 85% yield but with only 54% D incorporation.^32^ Second, **3** can be prepared via Ni-catalyzed methylation with iodomethane-*d*_3_ in >69% yield on a multigram scale (% D not provided).^33^ Finally, Falb et al. demonstrated that **3** could be prepared on a multigram scale in 88% yield with >99% D enrichment using Suzuki–Miyaura cross-coupling conditions.^34^ Initially the deuteromethyl group was installed using expensive potassium methyl trifluoroborate (CD_3_BF_3_K). Despite the fact that this route enabled the product to be formed in 88% yield, however, the procedure was modified to avoid the use of this chemical by employing greener and less expensive CD_3_B(OH)_2_ (Scheme 1). The reaction could be performed by commencing with **8** and proceeding either via: (a) the “methylation-last” route (involving coupling of methylboronic acid-*d*_3_ with **9** using Pd(OAc)_2_ and the RuPhos ligand) in 84% overall yield, or (b) the “methylation-first” route (**8** → **10**→ **11**→ **3**).

Regrettably, for the latter, the percent yield for the Chan–Lam conversion **11** → **3** was not provided. We can assume it is similar to the yield obtained for the Chan–Lam coupling reaction of the nondeuterated analogue of **11** with phenyl boronic acid (70%)^34^ giving an overall yield for the “methylation first” route of 45%.

#### RNA-Dependent RNA Polymerase (RdRp) Inhibitors

The enzyme RNA-dependent RNA polymerase (RdRp) is an important therapeutic target in RNA virus-caused diseases, including SARS-CoV-2. Nucleoside inhibitors (typically in their nucleoside triphosphate form) act by binding to the RdRp protein at the enzyme active site, therefore interfering with the RNA synthesis step.^35,36^ In this short subsection, we describe deuterated RdRp inhibitors that have been repurposed for the potential treatment of COVID-19.

##### Deuterated Oral Remdesivir Derivative VV116.

**Figure 3:**
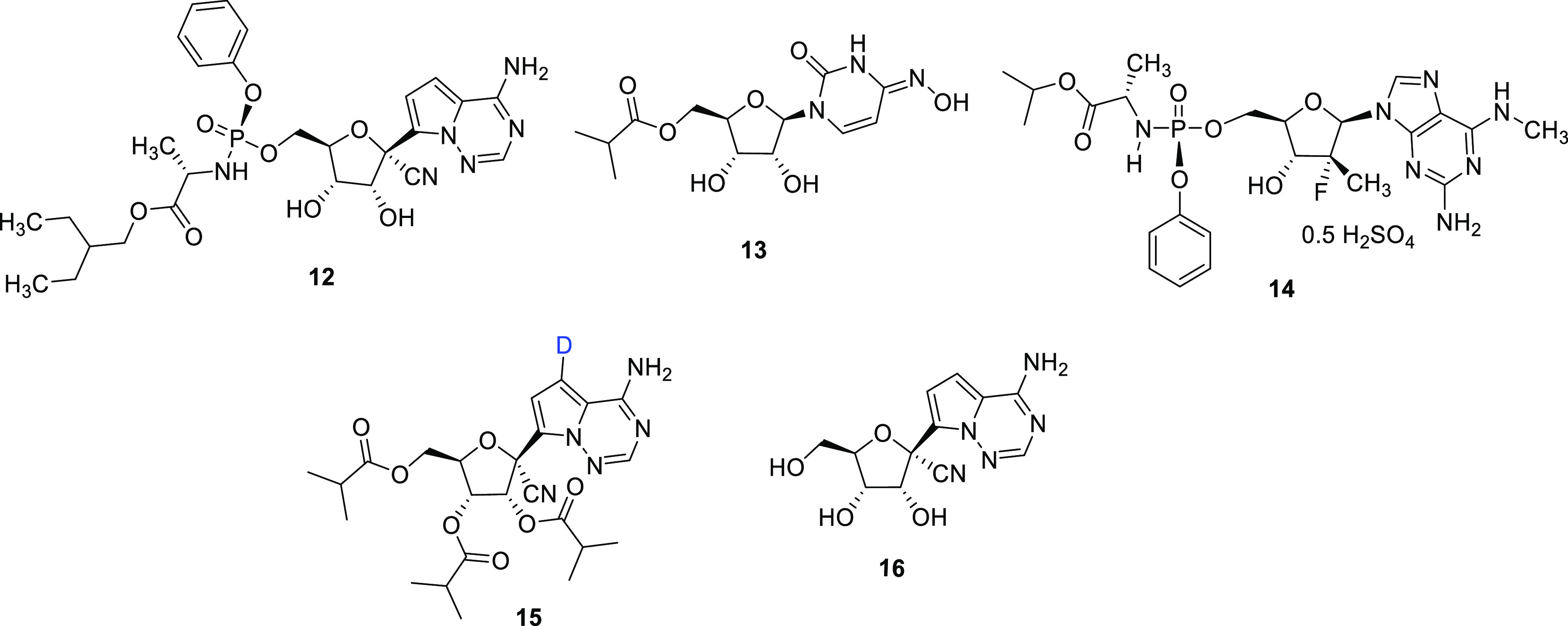
Chemical structures of RdRp inhibitors **12**–**16**.

 Remdesivir (**12**) (Figure 3), an intravenously administered nucleotide prodrug, is currently approved for treatment of COVID-19 by the FDA.^5^ Two other analogs [molnupiravir (**13**) and AT-527 (**14**)], taken orally, are in phase II/III clinical studies for COVID-19 [ClinicalTrials.gov identifiers NCT04405570 (molnupiravir) and NCT04709835 (AT-527)].^37^ Xie et al. have since developed a deuterated oral anti-SARS-CoV-2 nucleoside candidate, VV116 (**15**), also under clinical evaluation (phase II/III) as a COVID-19 therapeutic agent (ClinicalTrials.gov Identifier NCT05242042).^38^ Compound **15**, deuterated at C7 of the pyrrolotriazine ring, is a modified version of GS-441524 (**16**), the parent nucleoside of remdesivir (**12**), which inhibits the replication of SARS-CoV-2 but mainly targets the liver, whereas COVID-19 is primarily a lung disease.

Deuteration at this position is hypothesized to inhibit enzymatic degradation of the ring (either by oxidation of the double bond or by ring opening of the triazine).^38^ In addition, the inclusion of a tri-isobutyrate ester functionality in **15** improved the in vivo pharmacokinetics compared with that of the parent nucleoside (**16**), while its formulation as the hydrobromide salt gave it extra water solubility (and other enhanced physical properties). Compared with other similar derivatives of **16**, **15** showed the most favorable physicochemical properties and had superior oral bioavailability, anti-SARS-CoV-2 efficacy, and safety in mice, rats, and dogs in the subsequent preclinical study. Furthermore, a recent study demonstrated the safety and tolerability of **15** in healthy Chinese patients.^39^

**Scheme 2:**
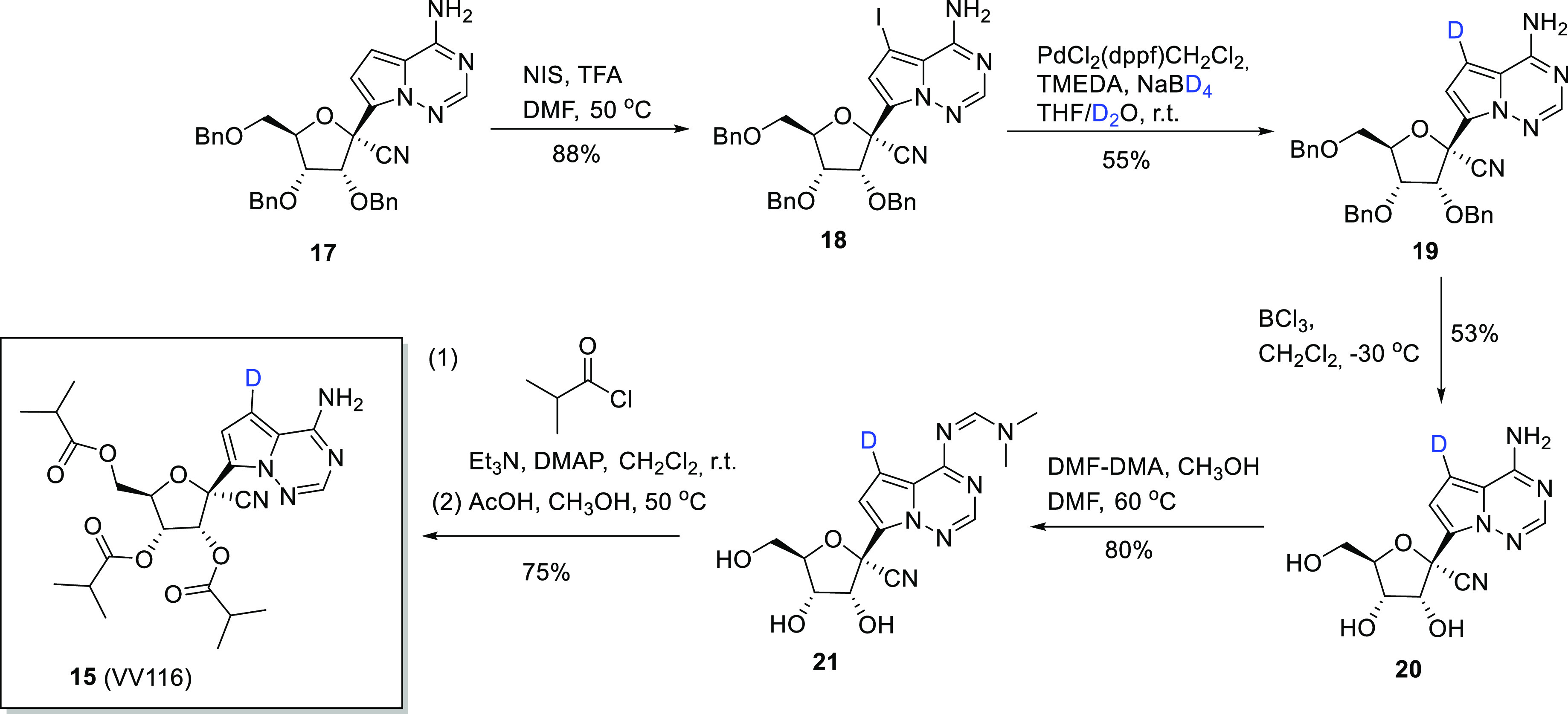
Synthetic Pathway to VV116 (**15**) Commencing with Nondeuterated Substrate **17** Adapted with permission from ref (38). Copyright 2021 Springer Nature.

VV116 (**15**) can be prepared in 15% overall yield in 5 steps commencing with the commercially available substrate **17** (CAS No. 1355357-49-1) (Scheme 2).^38^ Compound **17** is initially iodinated to form intermediate **18** before deuterium is introduced into **18** using a palladium catalyst complexed with TMEDA and reduced using NaBD_4_ to form **19** in 55% yield (≥98% D incorporation). **19** could then be transformed into **15** via a further three steps: OH deprotection to **20** (monodeuterated analogue of **16**), amine protection to form **21**, and, finally, a one-pot acylation followed by amine deprotection to **15**.

In a subsequent study,^40^ Zheng et al. describe a more efficient approach to the synthesis of **19** which involves reducing **18** with triethylamine, palladium, and deuterium gas (D_2_) at 60 °C for 1 h. In this method, **19** was produced in 92% yield with D incorporation of 99%. In addition, Zheng and co-workers reported the synthesis of deuterated analogues of GS-441524 (**20** and **20a**–**d**) along with a comparison of their efficacy against SARS-CoV-2 in vitro against nondeuterated GS-441524 (**16**) (Table 1). Essentially, all of the deuterated GS-441524 analogs demonstrated similar antiviral activity to GS-441524 against SARS-CoV-2. A further comparison of the metabolic profiles and/or IC_50_ values of **16** with **20** and **20a**–**d** would add value to the study.

**Table 1:**
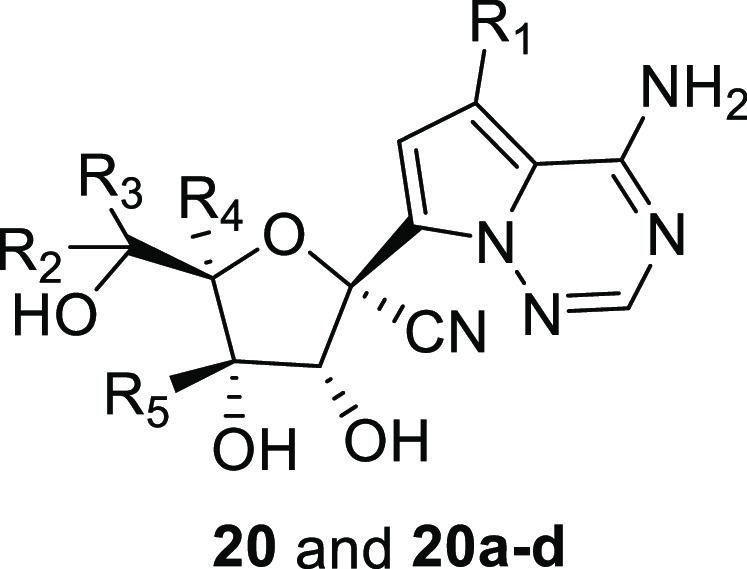
Inhibition of SARS-CoV-2 Replication and Cellular Toxicity by Deuterated Remdesivir Analogues **20** and **20a**–**d** In Vitro (in Vero E6 cells) Relative to Nondeuterated Remdesivir Nucleoside **16**^a^

compound	R_1_	R_2_	R_3_	R_4_	R_5_	EC_50_ (μM)	CC_50_ (μM)
**16**	H	H	H	H	H	0.33	>100
**20**	D	H	H	H	H	0.24	>100
**20a**	H	D	D	H	H	0.25	>100
**20b**	D	D	D	H	H	0.23	>100
**20c**	D	D	D	D	D	0.23	>100
**20d**	H	D	D	D	D	0.31	>100

a Adapted with permission from ref (40). Copyright 2022 Elsevier.

##### Deuterated Thymine Analogue.

**Scheme 3:**
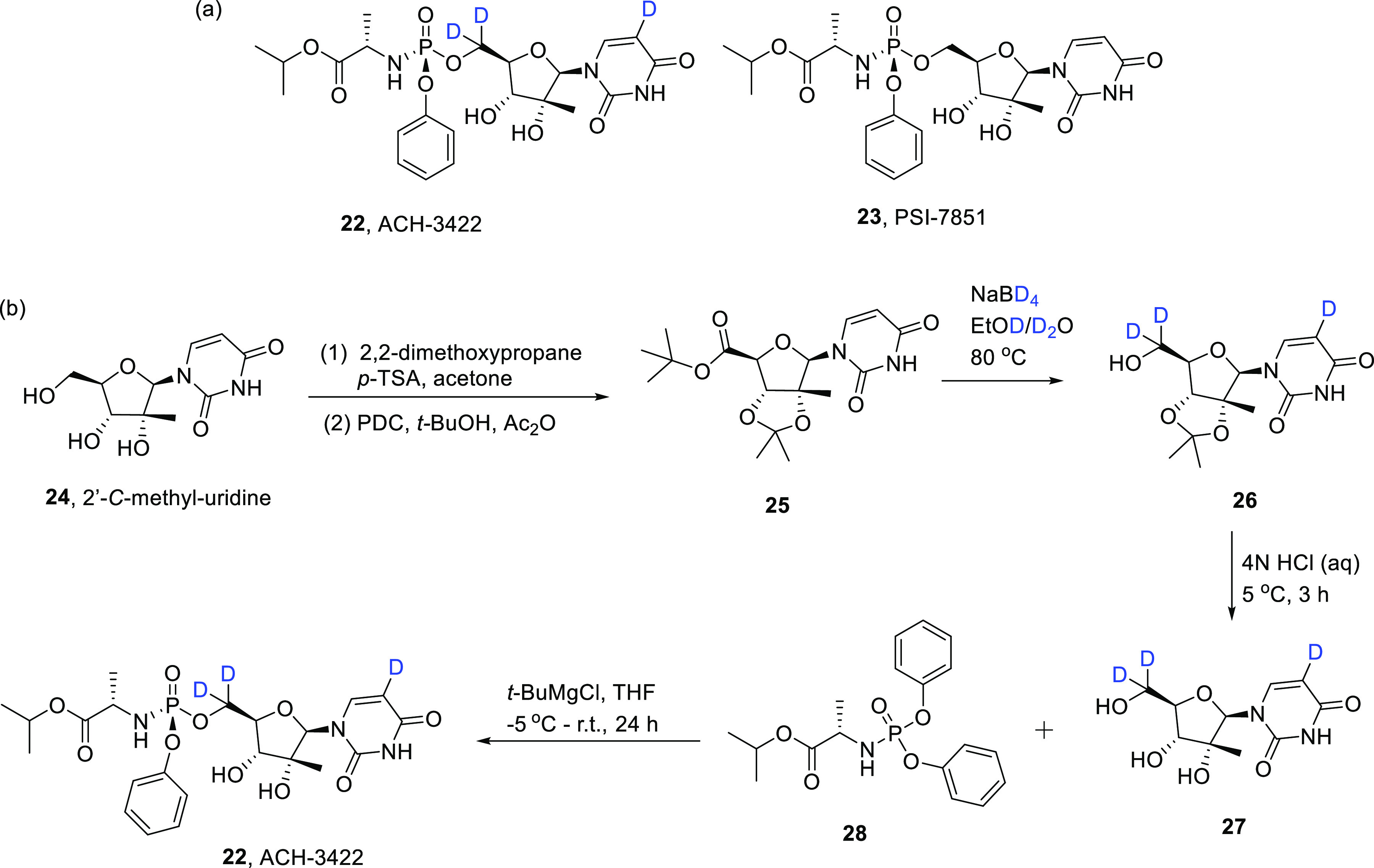
(a) Chemical Structures of ACH-3422 (**22**) and the Parent Drug PSI-7851 (**23**); (b) Synthesis of ACH-3422 by Initial Preparation of Deuterated Acetonide **26** Followed by a Double OH Deprotection to **27**, which Then Reacts with **28** To Form the Deuterated Thymine Analogue **22**

 ACH-3422, CAS No. 798779-31-4 (abbreviated **22**) [(Scheme 3a), the deuterated analogue of PSI-7851 (**23**), is also an RNA-dependent RNA polymerase and has been considered for the treatment of COVID-19.^35,41^**22** contains three deuteriums: one on the pyrimidine and two on the ribose group side chain. Substituting hydrogen for deuterium at these positions was proposed to improve the safety profile of the parent drug **23** by enabling a more stable drug concentration and reducing the production of toxic metabolites. Indeed, in a separate study, **22** was well tolerated and did not induce any serious adverse events in both healthy volunteers and hepatitis C patients.^41^ Importantly, among COVID-19 patients, increasing the dose of **22** resulted in increased viral decline, and viral clearance was achieved in 50% of patients after the administration of 700 mg/day over 2 weeks.^35^

The preparation of **22** commences with the initial synthesis of deuterated acetonide **26** by a combined reduction–deprotection and H/D exchange at the α-C of acetonide ester **25** (Scheme 3),^42^ itself prepared from commercially available 2′-*C*-methyl-uridine, **24** (CAS No. 31448-54-1).^43^ Although an initial ^1^H NMR spectroscopic analysis of **26** indicated ∼85% deuterium incorporation at the 5-uracil position, this was increased to >98% by filtration, removal of EtOD, and addition of more D_2_O, followed by reheating of the resulting mixture at 95 °C.^42^ Subsequent deprotection of **26** with aqueous HCl provided nucleoside **27**, which was then reacted with **28** under Grignard conditions to form **22** (regrettably, no yields were reported for the reaction procedure).^42,43^

#### Deuterated Dexamethasone

Dexamethasone (**29**), a corticosteroid and anti-inflammatory agent, has demonstrated efficacy as a treatment for COVID-19 patients.^44^ Thus, the deuterated analogue^45^ is in demand—both as an internal MS standard and to test as a therapeutic agent with improved bioavailability and safety profile (compared with dexamethasone). As a glucocorticoid, long-term use of dexamethasone has the potential to produce a wide range of undesirable effects, many mediated by the glucocorticoid receptor which regulates the expression of a vast array of genes. When used for relatively short periods (up to 2 weeks), dexamethasone is not usually expected to produce serious toxicities^46^ although higher doses can produce neurological effects, stomach ulcers, autoimmune and cardiovascular events, and pancreatitis.^44,47^ Thus, lowering the dosage while maintaining the necessary bioavailability of the corticosteroid by regulating the metabolism could offer a safer option for the use of dexamethasone for COVID-19 as well as in treating other conditions. In a similar fashion to the other drugs we have discussed, deuteration could enable this by hindering the metabolism of dexamethasone.

**Scheme 4:**
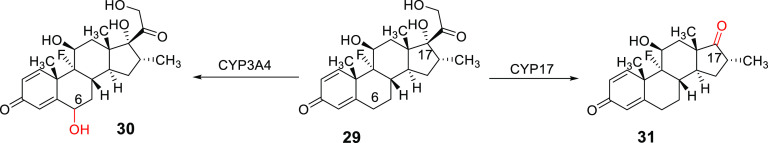
Metabolic Profile of Dexamethasone (**29**)

Furthermore, studies involving comparisons between dexamethasone and its deuterated analogues could provide valuable information regarding the role of the metabolites in instigating adverse side effects (not yet studied, as far we can ascertain), which could remove any uncertainty regarding its role as a therapeutic agent for COVID-19. The main routes of metabolism of dexamethasone (**29**) involve hydroxylation at C-6 (by CYP3A4 enzymes) and replacement of the 2-hydroxyethan-1-one with a ketone group at C-17 (by CYP17), resulting in the metabolites **30** and **31**, respectively (Scheme 4).^48^

**Scheme 5:**
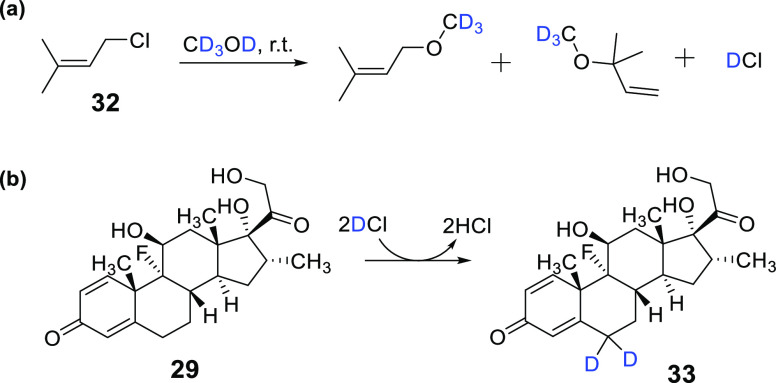
(a) Generation of DCl from Reaction of Prenyl Chloride **32** with Methanol-*d*_4_ Followed by (b) Acid-Catalyzed H/D Exchange of Dexamethasone (**29**) at C-6 (a) Adapted with permission from ref (49). Copyright 2021 Royal Society of Chemistry.

The synthesis of a deuterated version of **29** was first reported in 1997 by Best et al., although the exact positions of H/D exchange were not given.^45^ More recently, Darshana et al. reported the H/D exchange of dexamethasone at C-6 based on the in situ spontaneous generation of deuterium chloride (DCl) from a prenyl chloride (**32**) under mild conditions (rt, 48 h) in CD_3_OD (Scheme 5a).^49^ The generated DCl induced H/D exchange within the dexamethasone at the α and γ positions next to the carbonyl groups of **29** via acid catalysis chemistry, resulting in 74% deuterium incorporation in **33**, produced in 98% yield (Scheme 5b).

### New Drugs

#### Deuterated Arachidonic Acid Ethyl Ester

**Scheme 6:**
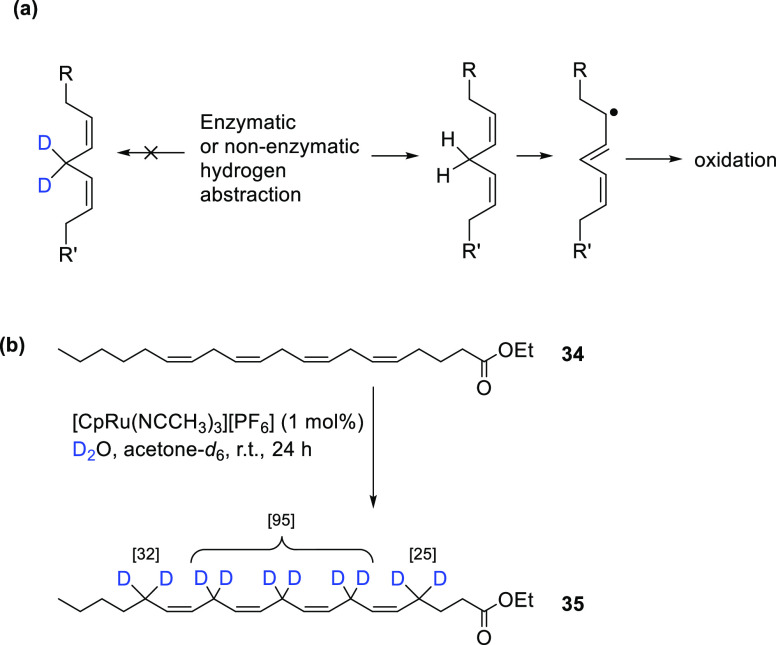
(a) Hydrogen Abstraction of a Bisallylic Hydrogen, Where the Key Step of PUFA Oxidation Is Inhibited by Deuteration; (b) Synthesis of the Ethyl Ester of Arachidonic Acid-*d*_6_ (**35**) from the Nondeuterated Analogue (**34**) Adapted with permission from ref (51). Copyright 2022 MDPI.

The ethyl ester of arachidonic acid (**34**) (Scheme 6) is a major component of lipid bilayers and the key substrate for the eicosanoid cascades. **34** is initially hydrolyzed to the acid form by the enzyme phospholipase A2, prior to enzymatic oxidation, e.g., by cytochrome P450 enzymes, and the metabolite products induce inflammatory responses in nearly all tissues, including lung tissues. Oxidation products of the acid form of **34** are elevated in COVID-19 patients.^50^ Thus, one strategy to interfere with the metabolism is by deuteration at the point of oxidation (Scheme 6a). Molchanova et al. demonstrated that deuteration at the bisallylic positions within **34** to form **35** (Scheme 6b) substantially decreases the overall rate of oxidation when hydrogen abstraction is an initiating event.^51^ The researchers also found that oral dosing with **35** resulted in successful incorporation of **35** into various tissues and significantly reduced *E. coli* lipopolysaccharide (LPS)-induced adverse effects in the lung area. This work therefore suggests novel therapeutic avenues for reducing lung damage during COVID-19 infection.

The ethyl ester of arachidonic acid-*d*_6_ (**35**) can be synthesized from the naturally occurring, nondeuterated ethyl ester of arachidonic acid (**34**) using a ruthenium catalyst (1 mol %) in quantitative yield,^52^ as shown in Scheme 6. The degree of deuteration was reportedly 32% and 25% at the monoallylic and 95% at the bisallylic positions of **35**, respectively.

Deuteration at the monoallylic sites may not affect drug potency since oxidation occurs predominantly at the bisallylic sites.^53^ Furthermore, one study suggests that the addition of polyunsaturated fatty acids (PUFA) deuterated at the monoallylic sites does not protect the cells from oxidation.^54^ Nevertheless, further studies might show the efficacy or toxicity of deuterium incorporation at the monoallylic sites. In addition, PUFAs with only partially deuterated bisallylic positions seem to be protected the same as that of PUFAs with completely deuterated bisallylic positions. Moreover, from the same study, we can conclude that inclusion of just a fraction of deuterated PUFAs (20–50%) in the total pool of PUFAs appears to preserve mitochondrial respiratory function and confers cell protection. Thus, a quantitative study involving samples of deuterated arachidonic acid to varying degrees in preserving mitochondrial function might provide clarification around this aspect.

#### Deuterated Broad-Spectrum Inhibitors of SARS-CoV-2 3CL Proteases

**Figure 4:**
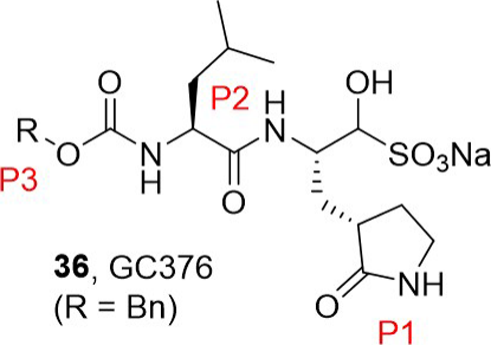
Chemical structure of 3CL^pro^ inhibitor GC376 (**36**). P1, P2 and P3 are the fragments of the inhibitor known to bind to the active site of M^pro^ (the protease that is key to the replication of SARS-CoV-2).

Dampalla et al.^55^ developed the dipeptidyl inhibitor GC376 (**36**) (Figure 4) in which the P1, P2, and P3 residues are: a lactam-containing glutamine surrogate, leucine, and a benzyl acetate, respectively. P1, P2 and P3 refer to fragments of the inhibitor that target the virus proteases of SARS-CoV-2 by binding to the active site of MERS-CoV 3-chymotrypsin-like protease (3CL^pro^), the protease that is central to the replication of SARS-CoV-2 (generally known as the coronavirus main protease, M^pro^).^55^ The potential to achieve improved binding interactions was identified by introducing different functionalities at the carbamate R groups in the inhibitors. These are able to dock within an S4 pocket of 3CL^pro^ surrounded by a set of primarily hydrophobic residues.

**Scheme 7:**
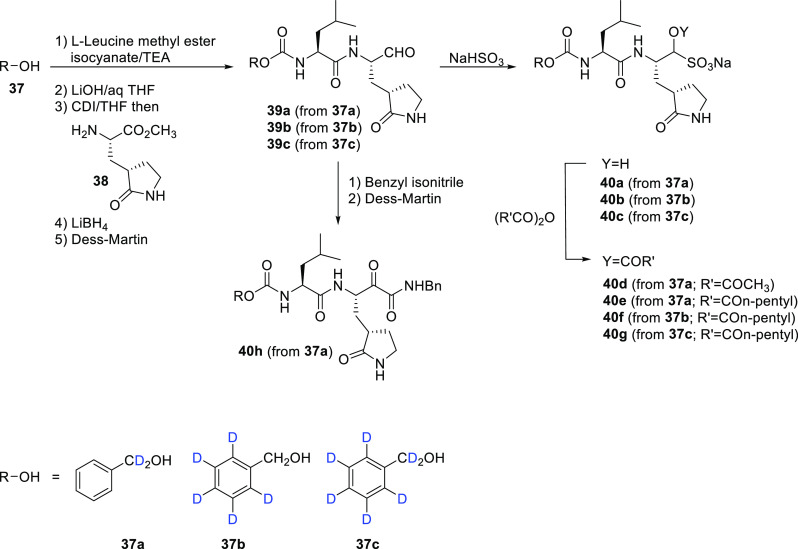
Synthesis of Deuterated GC376 Derivatives **39**–**40** As Described by Dampalla et al.^55,56^

It was considered that deuterated variants of GC376 might possess some superior properties as therapeutic agents compared to the corresponding nondeuterated GC376 drug, such as improved pharmacokinetics, lower toxicity, and higher efficacy. Eleven deuterated variants of GC376 were therefore studied by replacing hydrogen with deuterium at the metabolically vulnerable sites of GC376 (the carbamate R groups, the aromatic ring, and the benzylic carbon).^55^ The structures of the deuterated variants of GC376 are shown in Scheme 7 (compounds **39a**–**c** and **40a**–**h**). They were synthesized using a reaction sequence previously employed for the synthesis of nondeuterated analogues. Briefly, deuterated benzyl alcohols **37a**–**c** (purchased) were reacted with l-leucine isocyanate methyl ester to yield carbamate derivatives, which were then hydrolyzed to the corresponding acids with lithium hydroxide (Scheme 7). The subsequent coupling of the acid to the glutamine surrogate methyl ester **38** into dipeptides followed by lithium borohydride reduction and oxidation with Dess–Martin periodinane reagent yielded aldehydes **39a**–**c**. The bisulfite adducts **40a**–**c** were generated by the treatment of **39a**–**c** with sodium bisulfite. Further treatment of **40a**–**c** with acetyl or *n*-pentyl anhydride resulted in the corresponding esters **40d** and **40e**–**g**, respectively. Reaction of **39a** with benzyl isonitrile (benzyl isocyanide) with subsequent Dess–Martin oxidation afforded compound **40h**. Regrettably, no % D values were provided by the authors for the deuterated compounds shown in Scheme 7, although these are likely to be the same as the commercially obtained alcohols (**37a**–**c**).

Crystal structure investigations revealed that deuteration did not alter the interactions between the deuterated GC376 (**40a**) and the 3CL^pro^. However, the deuterated variants showed enhanced activity, and this was attributed to tighter binding to the target or improved physicochemical properties of the drug.^55^ The presence of the aldehyde group in **39a**–**c** is associated with toxicity; hence, the inclusion of deuterium was also meant to reduce the toxicity of these derivatives.

**Figure 5:**
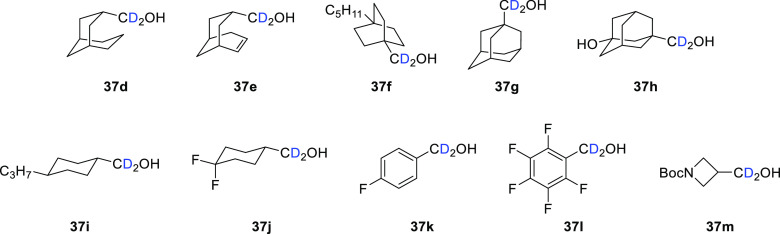
Chemical structures of deuterated alcohol substrates **37d**–**m** used in the synthesis of GC376 derivatives **39** and **40**.

The same authors prepared and evaluated another series of carbamate derivatives of GC376, deuterated on the alcohol side R commencing from the alcohol inputs **37d**–**l** (Figure 5). Synthesis of the inhibitors commencing from alcohols **37d**–**l** was via a separate process (not shown) involving initial treatment of the alcohols with *N*,*N*′-disuccinimidyl carbonate followed by coupling of the resulting mixed carbonate to a Leu-Gln surrogate amino alcohol to form aldehyde (analogous to **39a**–**c**) and bisulfite (analogous to **40a**–**c**) products.^56^ Compounds **37j**–**l** led to fluorinated and deuterated GC376 derivatives. All analogues were prepared to test whether inclusion of fluorine or deuterium might improve the potency, physicochemical parameters, and pharmacokinetics of the inhibitors compared with the corresponding nondeuterated inhibitor.

**Figure 6:**
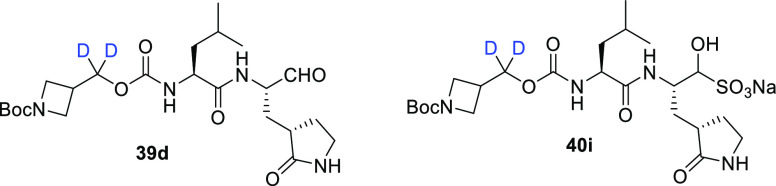
Chemical structures of azetidine-containing inhibitors **39d** and **40i**.

In addition, aldehyde **39d** (analogous to **39a**–**c**) and bisulfite **40i** (analogous to **40a**–**c**) were prepared by following this reaction sequence from azetidine alcohol **37m** (Figures 5 and 6).^57^ The azetidine cap, along with a series of spirocyclic analogues, was investigated to potentially exploit the new active site of the protease. The effect of deuteration on pharmacological activity was investigated by determining the IC_50_ values against SARS-CoV-2 3CL^pro^ (0.33 μM for **39d** and 0.34 μM for **40i**) and comparing these with those of the corresponding nondeuterated analogues (0.41 and 0.50 μM for nondeuterated for **39d** and **40i**, respectively). The authors anticipate that deuterated variants of similar inhibitors will likely display improved pharmacokinetics in future studies.

**Scheme 8:**
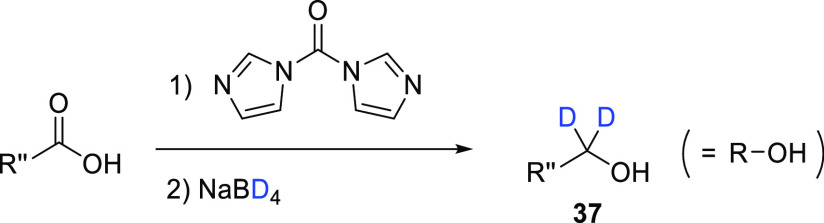
Generic Schematic Showing Preparation of Deuterated Alcohols (**37i**–**l**) from Commercially Available Carboxylic Acid Precursors

Alcohols **37d**–**h** were purchased, while **37i**–**l** were obtained by treatment of the commercially available precursor carboxylic acid with carbonyl diimidazole followed by the addition of NaBD_4_ (general synthesis shown in Scheme 8). No % D values were reported for any of the synthesized alcohols, but it could be assumed that the purchased deuterated alcohols had high (>98%) % D incorporation which would have been carried through to the final products. An alternative synthesis of α,α-dideuterio alcohols directly from feedstock carboxylic acids using D_2_O as the D source and avoiding pyrophoric alkali metal deuterides such as NaBD_4_ was reported by Szostak et al.^58^ This reaction proceeds after the activation of Sm(II) with a Lewis base and results in excellent levels of % D incorporation (85–96 D_2_) across a wide range of substrates.

**Scheme 9:**
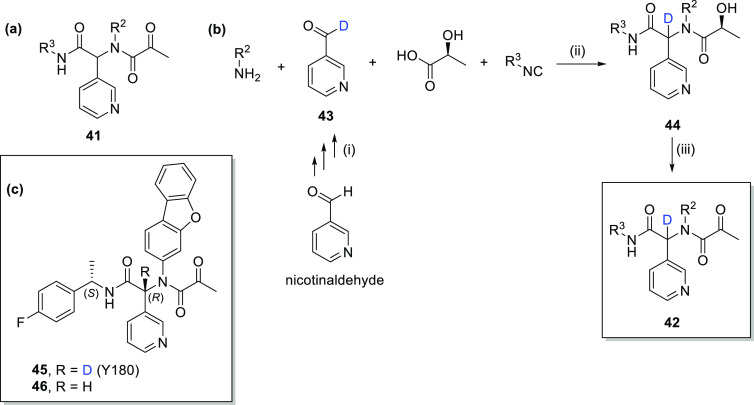
(a) General Chemical Structure of M^pro^ Inhibitor **41** Explored by Quan et al.;^61^ (b) Reaction To Form General Deuterated M^pro^ Inhibitor, **42**: (i) Formyl-Selective Deuteration of Nicotinaldeyde to **43**, (ii) Classical One-Pot Ugi-4CR To Form Diamine Derivative **44**, and (iii) Dess–Martin Oxidation of **44** to **42**; (c) Chemical Structures of **45** (Y180) and Its Nondeuterated Analogue **46**

As has already been pointed out, M^pro^ inhibitors are promising candidates for the treatment of COVID-19 because M^pro^ plays a crucial role at the onset of viral replication. Furthermore, M^pro^ is conserved among various variants of concern. Thus, any interruption of its catalytic activity could represent a relevant strategy for the development of anticoronavirus drugs. However, the majority of M^pro^ inhibitors belong to a class of compounds known as “peptidomimetics” (synthetic molecules designed to mimic the structural domain within a natural protein^59^); thus, they often possess poor pharmacokinetic properties and oral bioavailability.^60,61^ To counter this, Quan et al. developed a series of orally available M^pro^ inhibitors with potent in vivo antiviral activity against emerging variants of SARS-CoV-2.^61^ The general structure of the inhibitor (**41**) is shown in Scheme 9a. The various fragments (α-ketoamide, pyridine, R^1^, and R^2^) occupy the main four pockets of M^pro^. Due to two stereocenters in **41** (with a fixed *S*-configuration in 1-(4-fluorophenyl)ethan-1-yl substituent R^3^), the molecules are mixtures of epimers in which the (*R*)-epimers displayed much higher potency than the corresponding (*S*)-epimers, while the most active (*R*)-epimers rapidly convert to the less active epimer (*S*) in vivo, likely due to the presence of an exchangeable hydrogen in the chiral carbon center linking the two amides. Thus, to prevent or reduce configuration conversion, the authors incorporated deuterium at this position, forming deuterated M^pro^ inhibitors with general structure **42** (in addition to nondeuterated inhibitors **41**). The deuterated inhibitors were prepared using an Ugi 4-component reaction (Ugi-4CR). This involved the fusion of (*S*)-2-hydroxypropanoic acid, nicotinaldehyde-formyl-*d*_1_ (**43**), an amine (R^1^-NH_2_), and an isocyanide (R^2^-CN) to generate diamine derivative **44**, which was then converted to the general inhibitor (**42**) by a Dess–Martin oxidation [Scheme 9b(ii)]. Compound **43** can be generated from the nondeuterated nicotinaldehyde by a repeated reduction with NaBD_4_ (to the deuterated pyridin-3-ylmethanol) followed by Dess–Martin oxidation to the aldehyde three times, sufficient to generate compound **43** with D incorporation > 98% [Scheme 9b(i)]. We note that Dong et al.^62^ reported an alternative single-step route to **43** in 82% yield (94% D incorporation) that is formyl selective, uses readily available and safe D_2_O, and involves combined hydrogen-atom transfer photocatalysis and thiol catalysis. Similarly, Geng et al.^63^ reported an alternative single-step route to substituted analogues of **43** using readily available and safe D_2_O under mild reaction conditions with uniformly high (>95%) levels of D incorporation.

The authors prepared 11 deuterated inhibitors along with numerous nondeuterated inhibitors.^61^ Although the generated compounds showed similar potency to the nondeuterated analogues, conversion from the (*R*)- to the (*S*)-epimer was substantially reduced. Of the 11 isotopologues prepared, Y180 (**45**) (Scheme 9c) proved to be the most effective among the tested inhibitors with the lowest rate of epimerization and an IC_50_ of 8.1 nM against SARS-CoV-2 M^pro^ (compared with ∼13.3 μM for its nondeuterated analogue, **46**). **45** protected against wild-type SARS-CoV-2, B.1.1.7 (Alpha), B.1.617.1 (Kappa), and P.3 (Theta) with EC_50_ values of 11.4, 20.3, 34.4, and 23.7 nM, respectively. Oral treatment with **45** displayed a remarkable antiviral potency and substantially ameliorated the virus-induced tissue damage in both the nose and the lung of B.1.1.7-infected K18-human ACE2 (K18-hACE2) transgenic mice. Furthermore, treatment of B.1.617.1-infected mice (lethal infection model) with **45** improved their survival rate from 0 to 44.4% (*P* = 0.0086). Importantly, **45** was also highly effective against the B.1.1.529 (Omicron) variant both in vitro and in vivo. This is a nice example where deuterium does not directly affect the metabolism of the molecule but instead acts to prevent epimerization, thus enabling the preparation of a single, more efficient epimer. This approach is also known as deuterium-enabled chiral switching (DECS) and is a powerful tool to yield chirally pure drugs from chemically interconverting racemates, often resulting in therapeutic agents with improved efficacy and stability and reduced toxicity.^11,64^

## Deuterated Drugs as Internal MS Standards

In general, internal MS standards are useful in applications in which the amount of an analyte of interest within a mixture (e.g., in urine, blood) varies or is reduced during a process, e.g., due to adsorption, but needs to be accurately quantified throughout the procedure By including an isotope-labeled standard of known concentration within the mixture, it is possible to provide a measure of control throughout the procedure by correcting for analyte losses, therefore ensuring the accuracy and precision of reported concentrations.^65,66^ Generally, most quantitative analytical methods rely on mass spectrometry-related techniques such as LC/MS assays. Isotopologues are the most practical to use as internal MS standards as they usually coelute with the parent compound chromatographically and ionize in the same manner as the parent compound during mass spectrometry. However, deuterated compounds may demonstrate unexpected results such as different retention times for the analyte and deuterated internal MS standard from the reversed-phase LC or different extraction recovery and loss of deuterium due to H/D exchange. This is especially true for standards containing more than six deuterium atoms or with the label directly neighboring a basic nitrogen atom.^67^ For this reason, ^13^C-, ^15^N-, or ^17^O-labeled compounds may be more appropriate than deuterium-labeled compounds.^65^ On the other hand, H is typically more abundant in individual compounds, and deuterium is usually more cheaply and easily incorporated (e.g., via late-stage deuteration^68^), so deuterated internal MS standards are of great interest.^65,67^ To successfully separate compounds and prevent “cross talk”, the amount of deuteration can be varied (M + 3 is a standard requirement for hydrocarbons).^67^ Sometimes naturally occurring isotopes of the analyte also contribute significantly to the signal of the internal MS standard. This becomes more apparent in isotopically rich compounds, such as those containing sulfur, chlorine, or bromine, compounds with higher molecular weight, and those at high analyte/internal MS standard concentration ratios.^69^ However, each case is different, and the choice of which isotope label (or alternative internal MS standard) to use requires judicious discernment. An important requirement in preparing deuterated internal MS standards is a very high level of deuterium incorporation at the positions of enrichment within the standard, so that it does not cause interference with the analyte. There are several commercially available prediction software packages available, any of which enable the user to find the sum formula of a compound to calculate the necessary added mass units to generate a suitable MS standard.

Several deuterated internal MS standards have been used and prepared to quantify new or repurposed drugs and their metabolites for the treatment of COVID-19.

### Repurposed Drugs

**Figure 7:**
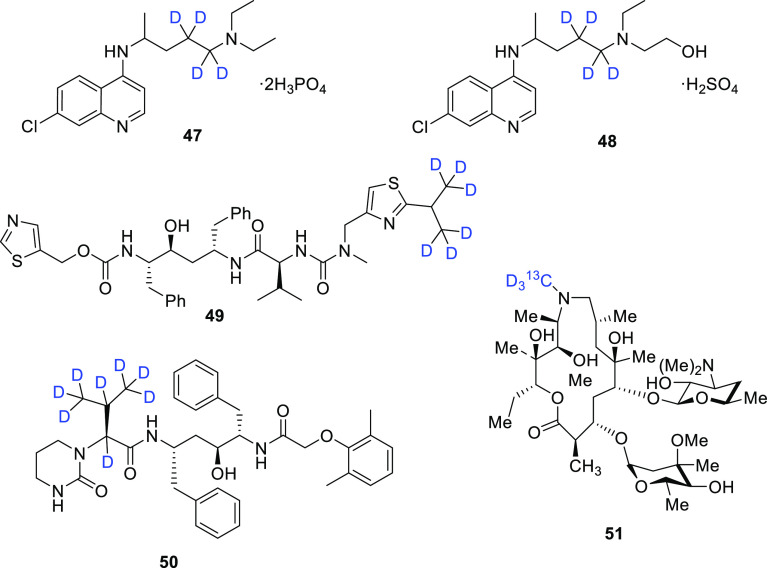
Chemical structures of internal MS standards **47**–**51**.

First, Habler et al. developed and validated a two-dimensional isotope-dilution liquid chromatography tandem mass spectrometry (ID-LC-MS/MS) method for the accurate and simultaneous quantification of drugs in human serum, specifically for quantifying several repurposed COVID-19 drugs simultaneously.^70^ The work was performed using stable deuterium-labeled analogues chloroquine-*d*_4_ phosphate (**47**), hydroxychloroquine-*d*_4_ sulfate (**48**), ritonavir-*d*_6_ (**49**), lopinavir-*d*_8_ (**50**), and azithromycin-^13^C-*d*_3_ (**51**) as internal MS standards (Figure 7), all commercially sourced. The dosage of repurposed COVID-19 therapeutics is typically derived from in vitro-generated half-maximum effective concentration (EC_50_) values for SARS-CoV-2 and pharmacology-based pharmacokinetic models from other diseases and clinical conditions. Because dosage regimens of repurposed drugs cannot always be suitably translated from their original purpose into appropriate drug exposure in COVID-19 patients due to pathophysiological alterations, it can lead to possible subtherapeutic or toxic concentrations without clinical benefit. In addition, polytherapy can result in unreliable drug levels due to interactions between different drugs. Therefore, therapeutic drug monitoring is crucial. The developed assay was designed to be an efficient method for the monitoring of these potential drug candidates in COVID-19 patients and to increase treatment efficacy and safety.^70^

**Figure 8:**
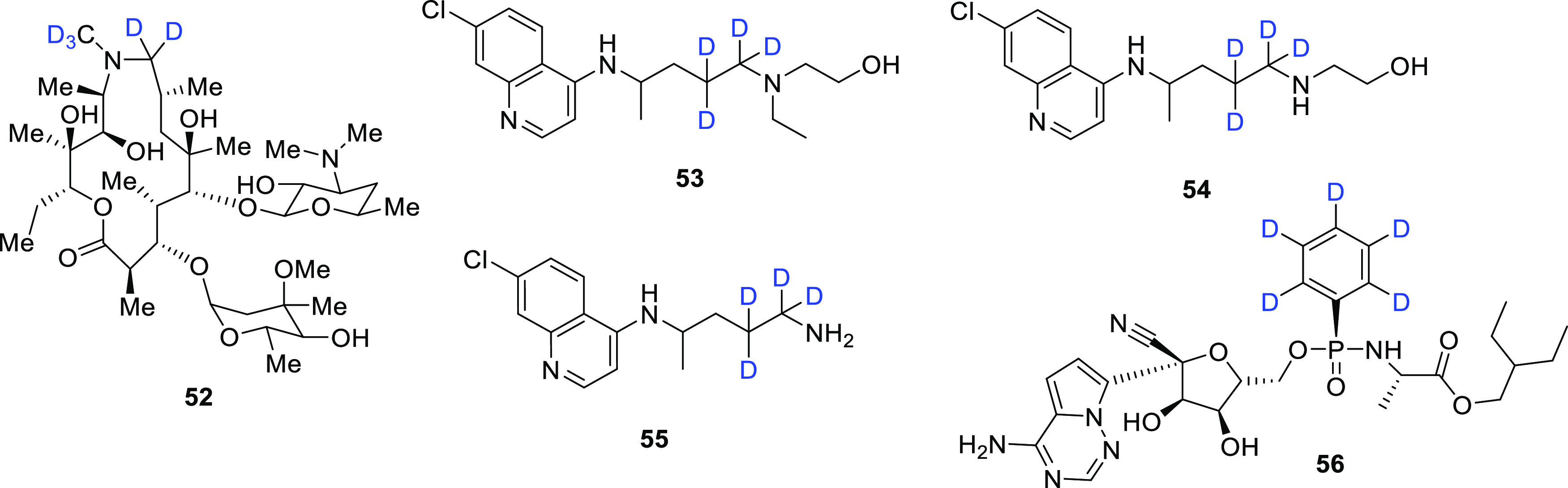
Chemical structures of internal MS standards **52**–**56**.

In a similar study, Sok et al.^71^ developed and validated the first ever LC-MS/MS method for simultaneous quantification of azithromycin, hydroxychloroquine (HCQ) (both antimalarial drugs and potential COVID-19 therapeutic agents), and two metabolites of HCQ, desethyl-HCQ and bisdesethyl-HCQ, in EDTA-treated human blood plasma. The study made use of the commercially available internal MS standards azithromycin-*d*_5_ (**52**), hydroxychloroquine-*d*_4_ (**53**) (the neutralized version of **48**), desethyl-hydroxychloroquine-*d*_4_ (**54**), and bisdesethylchloroquine-*d*_4_ (**55**) (Figure 8), and is suitable for clinical studies requiring a fast turnaround time and small sample volume (the assay requires only 20 μL of plasma). The method was developed to support clinical trials and to assess the pharmacokinetics and pharmacodynamics of these repurposed drugs in this new role.^71^

The FDA approval of the antiviral drug remdesivir for the treatment of COVID-19 in adult and pediatric patients 12 years or older requiring hospitalization led to the increased need for a simple, sensitive, and selective assay to quantify drug concentrations in clinical samples to study therapeutic dosing and provide pharmacokinetic studies. Therefore, Nguyen et al.^72^ developed and validated a rapid and sensitive LC-MS/MS assay for the quantification of remdesivir (compound **12**) in human plasma which made use of the commercially available deuterium-labeled analog remdesivir-*d*_5_ (**56**) (Figure 8) as the internal MS standard. This method has proven to be the most sensitive to date and is suitable for therapeutic dosing studies. The precision, accuracy, and selectivity all met the FDA Bioanalytical Guidelines.

**Figure 9:**
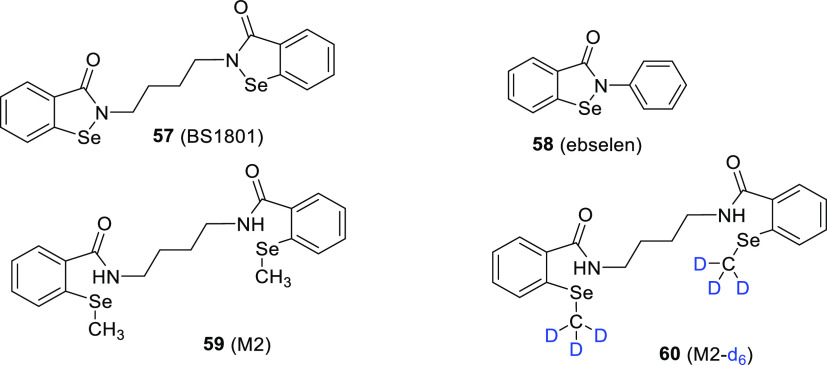
Chemical structures of ebselen (**58**) and its analogue (major metabolite of BS1801) **57**, along with **59** (M2) and its deuterated analogue **60** (M2-*d*_6_).

Tian et al.^73a^ reported the first-ever study carried out to identify and quantify the major circulating metabolites of BS1801 (**57**), an analogue of ebselen (**58**), found in the hepatocytes of different species and human plasma (Figure 9). Ebselen is one of the promising drugs for the treatment of COVID-19.^74^ To quantify the major BS1801 metabolite “M2” (**59**), an accurate and fast LC-MS/MS method was established which relied upon the use of a deuterated internal MS standard.

**Scheme 10:**
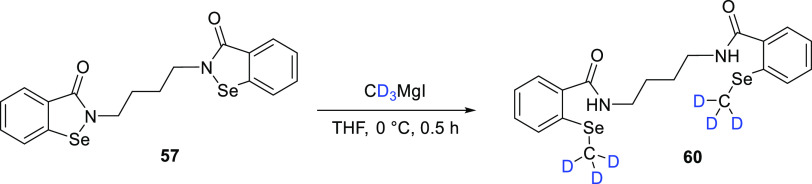
Synthesis of **60** from **57** via Grignard Chemistry Adapted with permission from ref (73). Copyright 2022 Elsevier.

To meet this need, the authors prepared a deuterated internal MS standard (M2-*d*_6_)^73b^ (**60**) by using a Grignard reagent to introduce a deuterated methyl group on the selenium atoms of **59** (Scheme 10). It was assumed that the final product had >99% D incorporation since this was the enrichment of the commercially obtained methyl-*d*_3_–magnesium iodide (CD_3_MgI) solution. The concept demonstrates the utility of late-stage deuteration. The analytical method was successfully used for the pharmacokinetic evaluation of BS1801 (**57**), demonstrating that this approach could provide a reference for the pharmacokinetic analysis of other selenium-containing drugs. The high D content of **60** is essential for the use of deuterated internal standards in LC-MS/MS analysis. Nevertheless, a greener alternative to traditional Grignard solvents might be implemented for the synthesis of **60** in the future.^75^

**Figure 10:**
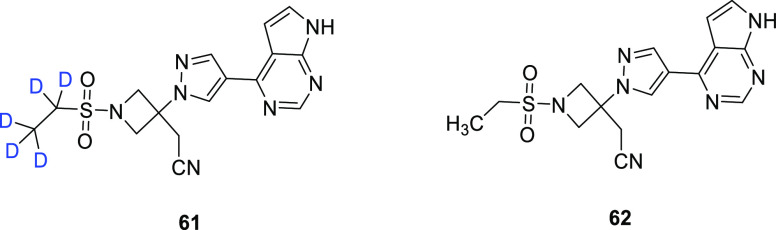
Chemical structures of baricitinib-*d*_5_ (**61**) and nondeuterated baricitinib (**62**).

**Scheme 11:**
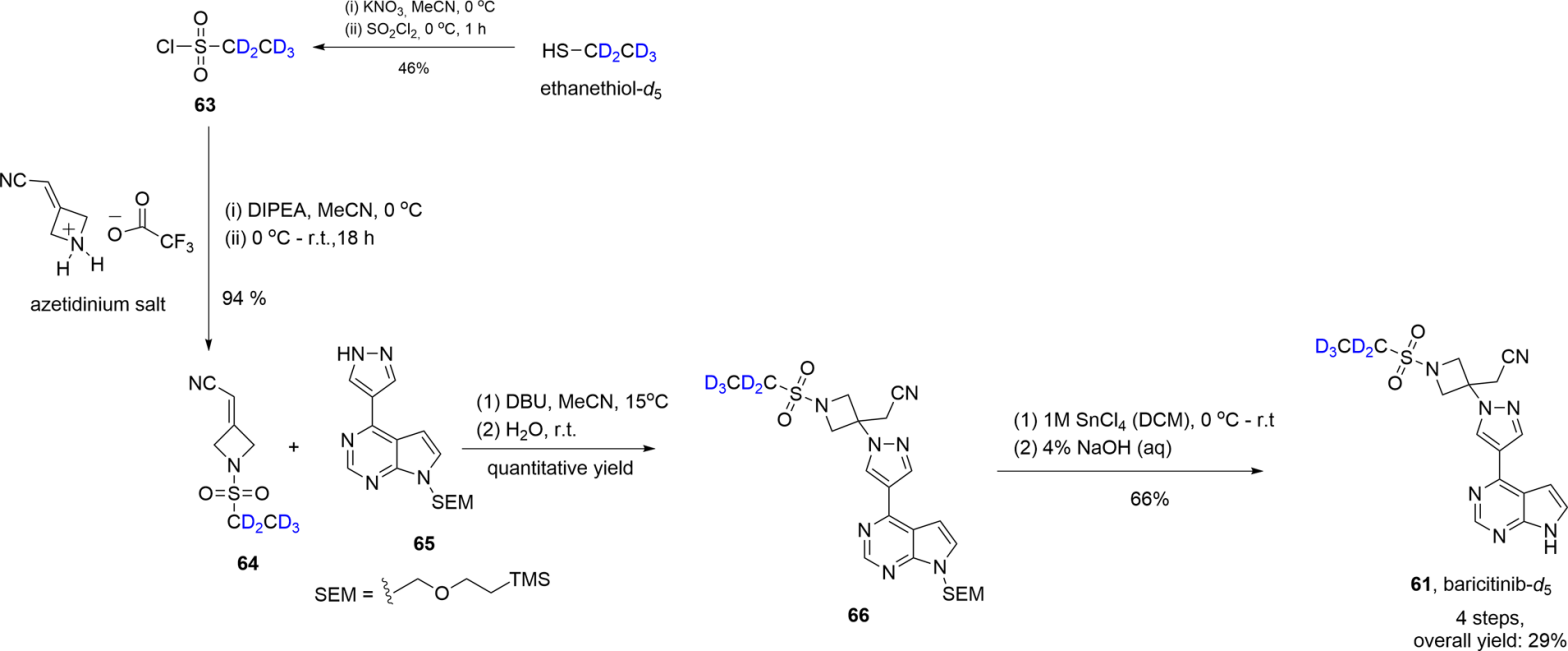
Synthesis of Deuterated Baricitinib (**61**) in a 29% Overall Yield, Starting from Commercially Available Ethanethiol-*d*_5_ Adapted with permission from ref (76). Copyright 2022 John Wiley & Sons, Inc.

Jansen-van Vuuren and Vohra^76^ reported the development of a simple synthetic route to baricitinib-*d*_5_ (**61**), the deuterated analog of baricitinib (**62**) (Figure 10). Baricitinib is a therapeutic agent used to treat rheumatoid arthritis and, as of May 11, 2022, approved by the FDA to treat COVID-19 in hospitalized adults requiring supplemental oxygen or ventilation.^77^ Prior literature describing synthetic pathways to **61** involved the use of toxic reagents, so the authors developed an alternative synthetic route, contingent on the initial integration of deuterium into the ethanesulfonyl component through the synthesis of ethanesulfonyl chloride-*d*_5_ (**63**) from commercially available ethanethiol-*d*_5_ (>98% D) (Scheme 11). **63** was immediately converted to the stable intermediate **64** in 94% yield upon reaction with an easily prepared azetidinium salt. **64** could then be converted to the desired product (**61**) in an additional two steps: reaction of **64** with commercially available **65** to form stable and isolable intermediate **66**, followed by trimethylsilylethoxymethyl (SEM) deprotection. The deuterated analog of baricitinib (**61**, 98% D), obtained in an overall yield of 29%, may be used as an internal MS standard in further studies.

### Deuterated Biomarkers as Internal MS Standards

Chhabra et al.^78^ in preparing heparin/heparan sulfate (HS) mimetics potentially targeted at a variety of diseases (including SARS-CoV-2), developed a fully quantitative LC-MS/MS assay for quantification of HS, also a biomarker for some lysosomal storage diseases, namely, the family of mucopolysaccharidosis (MPS) disorders.

**Scheme 12:**
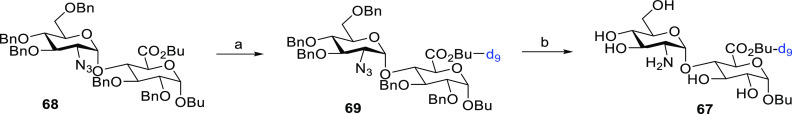
Synthesis of Deuterated Dissacharide **67** Reagents and conditions: (a) (i) 5 M NaOH/MeOH/CHCl_3_/H_2_O, rt, 48 h; (ii) *n*-BuI-*d*_9_, KHCO_3_, DMF, rt, 24 h; (b) 20% Pd(OH)_2_/C, MeOH, rt, 24 h. Adapted with permission from ref (79). Copyright 2019 American Chemical Society.

The disease causes the accumulation of undegraded HS which is associated with multiple pathologies in the brain and other organs. The published method proved to be efficient for the determination of HS in the brain tissue of mice with MPS. Given that quantification of HS in biological samples, e.g., urine or tissue, is complicated by its heterogeneity and high molecular weight, acid-based methanolysis or butanolysis resulting in desulfated disaccharide cleavage products which are detectable by a LC-MS/MS assay is a suitable alternative option. For this reason, the authors prepared a deuterium-labeled version (**67**) of the major HS disaccharide butanolysis product as an internal MS standard (Scheme 12). The synthesis involves the initial saponification of **68** with NaOH (aq) in a MeOH–chloroform solution.^79^ The crude carboxylate is then esterified under basic conditions with commercially available 1-iodobutane-*d*_9_ (≥98 atom % D) to give the disaccharide **69** in 86% yield. Hydrogenolysis then gave the deuterated disaccharide **67** in 57% yield (with the same % D as the 1-iodobutane-*d*_9_). The method may also prove useful in the study of HS mimetics intended for pharmaceutical purposes, including for drugs targeting COVID-19.^78,80^

### Deuterated Lipids as Internal MS Standards

**Figure 11:**
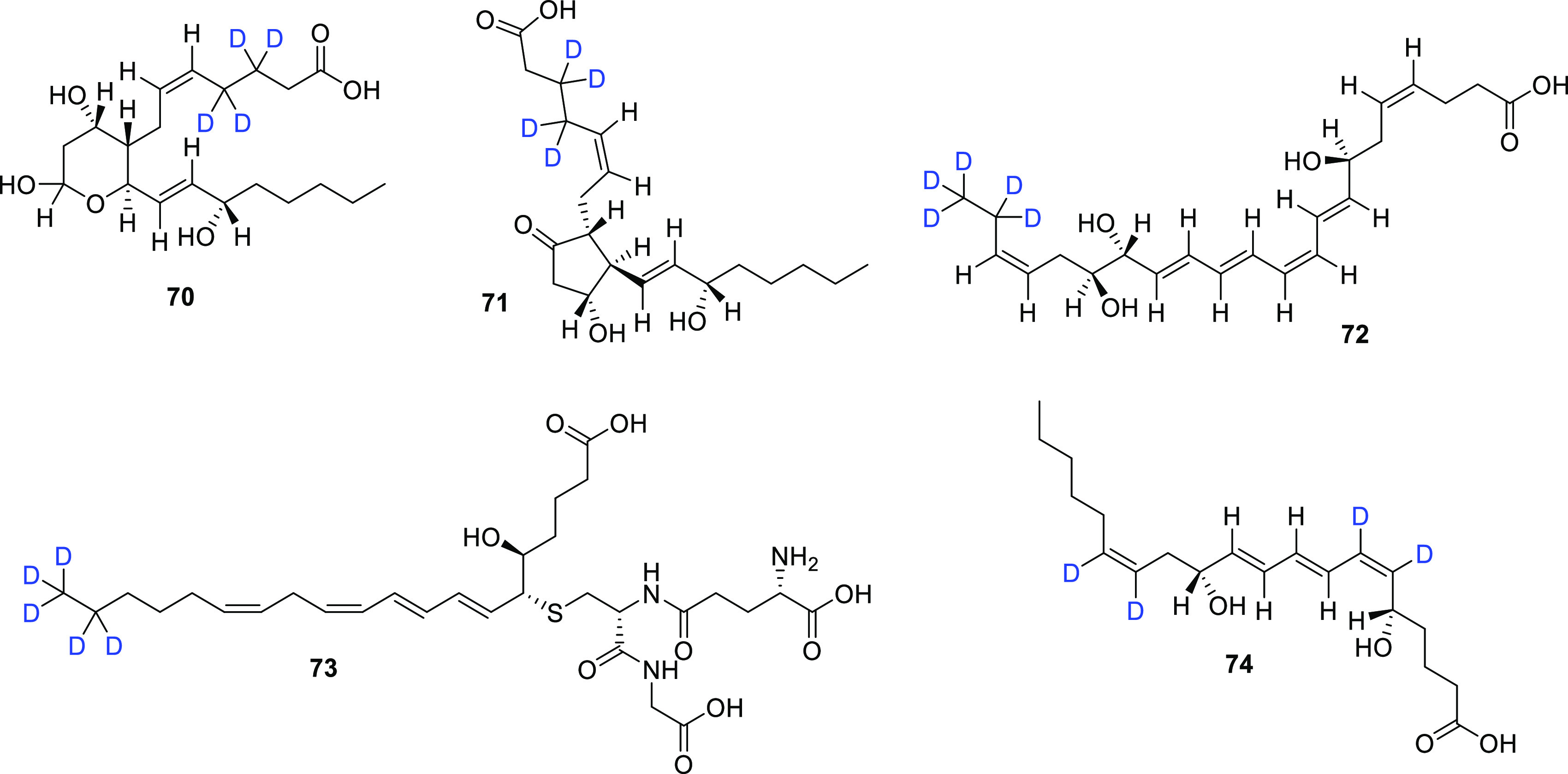
Chemical structures of commercially available deuterium-labeled lipids and lipid mediators **70**–**74**.

Selected bioactive lipids (BALs) and lipid mediators can initiate anti-inflammatory activity, including during acute lung inflammation and injury. As such, BALs are pharmaceutical targets in many inflammatory diseases, while higher levels of certain BALs might signal cases of severe COVID-19. Archambault et al.^81^ used commercially available deuterium-labeled lipids and lipid mediators (five examples (**70**–**74**) shown in Figure 11) as internal/surrogate standards in the LC-MS/MS quantification of certain BALs (eicosanoids and docosanoids) which modulate lung inflammation in severe COVID-19 patients. The goal was to find out if severe COVID-19 patients were characterized by increased BALs modulating lung inflammation. A targeted lipidomic analysis of bronchoalveolar lavages by tandem mass spectrometry on 25 healthy controls and 33 COVID-19 patients requiring mechanical ventilation was performed; indeed, an increase in fatty acids and inflammatory lipid mediators was observed. In the BALs of severe COVID-19 patients, a predominance of eicosanoids such as thromboxane B2 and prostaglandins was observed, which were quantified with the use of deuterated internal MS standards **70** (thromboxane B2-*d*_4_) and **71** (prostaglandin E2-*d*_4_) (Figure 11). An increase was also observed in d-series resolvins (pro-resolving mediators) and leukotrienes where deuterated internal MS standards **72** (resolvin D2-*d*_5_), **73** (leukotriene C4-*d*_5_), and **74** (leukotriene B4-*d*_4_) came into use.

Similarly, Barberis et al.^82^ used untargeted metabolomics and lipidomics analysis of plasma from COVID-19 patients and control groups to capture the host response to SARS-CoV-2 infection. A deuterated standard mix (Splash Lipidomix; https://avantilipids.com/product/330707) was used for LC-MS/MS detection of different lipid classes which act as potential COVID-19 biomarkers and therapeutic targets in plasma. It was found that several circulating lipids, triglycerides, and free fatty acids are correlated to the severity of the disease. The study also provided further evidence for considering phospholipase A2 (PLA2) activity as a potential factor in the pathogenesis of COVID-19 and a possible therapeutic target. A similar study^83^ focuses on patients from the Campania region, Italy.

This knowledge has the potential to assist in developing or repurposing drugs which could be helpful at modulating the observed lipidome to minimize the effects of pro-inflammatory lipids and enhance the effect of anti-inflammatory or pro-resolving lipid mediators.

## Conclusion and Future Opportunities

In conclusion, this review examines deuterated drugs which have been featured during the COVID-19 pandemic, either as potential therapeutic compounds or as internal MS standards for studying the pharmacokinetic or metabolic properties of new or repurposed COVID-19 drugs. Table 2 provides a summary of all deuterated compounds included in this review.

**Table 2:** List of Deuterated Drugs and Internal Standards Featured in This Review

code no.	name of drug	role played by deuterium	ref
deuterated drugs as therapeutics: repurposed drugs			
**3**	deupirfenidone (LYT-100)	inhibits the metabolism of the drug and enables less frequent dosing	Schmidt 2021^27^
			Liu and Dong 2012^30^
			Chen et al. 2021^29^
			Chen et al. 2022^28^
**15**	VV116	inhibits enzymatic degradation of the ring	Xie et al. 2021^38^
			Qian et al. 2022^39^
**16, 20**	GS-441524 deuterated analogues	might improve antiviral activity of GS-441524 against SARS-CoV-2	Zheng et al. 2022^40^
**22**	ACH-3422	improves the safety profile of the parent drug by enabling a more stable drug concentration and reducing the production of toxic metabolites	Gane et al. 2015^41^
			Tian et al. 2021^35^
**33**	dexamethasone-*d*_2_	might improve bioavailability and safety profile (hindered metabolism)	Darshana et al. 2021^49^
deuterated drugs as therapeutics: new drugs			
**35**	arachidonic acid-*d*_6_	decreases the overall rate of oxidation	Molchanova et al. 2022^51^
			Smarun et al. 2017^52^
**39, 40**	GC376 deuterated analogues	enhances activity due to tighter binding to the target or improves physicochemical properties of the drug	Dampalla et al. 2021^55−57^
**45**	Y180	deuterium-enabled chiral switch	Quan et al. 2022^61^
deuterated drugs as internal MS standards: repurposed drugs			
**47**–**51**	chloroquine-*d*_4_ phosphate	enables ID-LC-MS/MS quantification of repurposed COVID-19 drugs in human serum	Habler et al. 2021^70^
	hydroxychloroquine-*d*_4_ sulfate		
	ritonavir-*d*_6_		
	lopinavir-*d*_8_		
	azithromycin-^13^C-*d*_3_		
**52**–**55**	azithromycin-*d*_5_	enables LC-MS/MS quantification of repurposed drugs in EDTA-treated human blood plasma to support clinical trials and assess the pharmacokinetics and pharmacodynamics of this repurposed drug	Sok et al. 2021^71^
	hydroxychloroquine-*d*_4_		
	desethyl-hydroxychloroquine-*d*_4_		
	bisdesethylhydroxychloroquine-*d*_4_		
**56**	remdesivir-*d*_5_	enables LC-MS/MS quantification of remdesivir in human plasma	Nguyen et al. 2021^72^
**60**	M2-*d*_6_	enables LC-MS/MS quantification of the major BS1801 metabolite “M2”	Tian et al. 2022^73^
**61**	baricitinib-*d*_5_	could enable LC-MS/MS quantification of baricitinib	Jansen-van Vuuren et al. 2022^76^
deuterated drugs as internal MS standards: biomarkers and lipids			
**67**	deuterium-labeled HS disaccharide	enables LC-MS/MS quantification of HS	Ferro et al.^78−80^
**70**–**74**	thromboxane B2-*d*_4_	enables LC-MS/MS quantification of certain bioactive lipids	Archambault et al. 2021^81^
	prostaglandin E2-*d*_4_		
	resolvin D2-*d*_5_		
	leukotriene C4-*d*_5_		
	leukotriene B4-*d*_4_		

For practitioners of HIE, there are many avenues worth exploration.

First, the deuteration of repurposed or novel drugs being studied as therapy options for COVID-19 whose safety profile and/or bioavailability is poor or not yet be fully understood may be of interest. For example, although remdesivir has been approved for COVID-19 treatment, it can cause certain adverse side effects;^84^ thus, research into the safety profile of remdesivir using deuterated analogues (e.g., remdesivir-*d*_5_, **56**) could be of value.

Similarly, the synthesis of deuterated analogues of orally administered COVID-19 antiviral agents which are being considered for clinical trials, e.g., acriflavine,^85^ or which have advanced to late-stage trials, e.g., molnupiravir (NCT04405570)^86^ and Pfizer’s PF-07321332 (or paxlovid) (NCT05011513),^87^ would be useful as analytical standards and/or for a deeper understanding of the metabolic profiles of the nondeuterated versions.

Developing new synthetic methods to the drugs listed in Table 2 which involve mid- or late-stage deuteration is a welcome contribution to the field of HIE since this could provide a greener synthetic route by decreasing the number of steps and chemicals/resources needed.

Deuteration has been shown to stabilize drug enantiomers and epimers. This approach holds much potential for enabling the synthesis of pure enantiomers over racemic mixtures. However, there has been limited exploration in this area beyond basic research.

Arachidonic acid ethyl ester (**34**) metabolites are important mediators in many physiological and pathophysiological processes. In fact, many of the benefits and toxicities of both glucocorticoids and nonsteroidal anti-inflammatory drugs are due to blocking the production of beneficial and detrimental metabolites of **34**. Altering the metabolism of **34** via deuteration at specific points in the chemical structure could have a wide number of effects above and beyond what is presented in the section Deuterated Arachidonic Acid Ethyl Ester.

Overall, we hope that providing this reference tool and highlighting new and interesting avenues for deuteration is of value to isotope and medicinal chemists. We also anticipate that exposing different strategies for drug development and discovery would be beneficial in light of future global pandemic situations.
